# Direct control of store-operated calcium channels by ultrafast laser

**DOI:** 10.1038/s41422-020-00463-9

**Published:** 2021-01-19

**Authors:** Pan Cheng, Xiaoying Tian, Wanyi Tang, Juan Cheng, Jin Bao, Haipeng Wang, Sisi Zheng, Youjun Wang, Xunbin Wei, Tunan Chen, Hua Feng, Tian Xue, Keisuke Goda, Hao He

**Affiliations:** 1 School of Biomedical Engineering, Shanghai Jiao Tong University, Shanghai, 200030 China; 2 School of life science, the University of Science and Technology of China, Hefei, Anhui 230026 China; 3 Department of Physiology, School of Basic Medical Sciences, Anhui Medical University, Hefei, Anhui 230032 China; 4 Beijing Key Laboratory of Gene Resource and Molecular Development, College of Life Sciences, Beijing Normal University, Beijing, 100875 China; 5 Institute of Neurosurgery, Southwest Hospital, Army Medical University (Third Military Medical University), Chongqing, 400038 China; 6 Department of Chemistry, University of Tokyo, Tokyo, 113-0033 Japan; 7 Institute of Technological Sciences, Wuhan University, Wuhan, Hubei 430072 China; 8 Department of Bioengineering, University of California, Los Angeles, CA 90095 USA

**Keywords:** Biological techniques, Ion channel signalling, Calcium signalling

## Abstract

Ca^2+^ channels are essential to cell birth, life, and death. They can be externally activated by optogenetic tools, but this requires robust introduction of exogenous optogenetic genes for expression of photosensitive proteins in biological systems. Here we present femtoSOC, a method for direct control of Ca^2+^ channels solely by ultrafast laser without the need for optogenetic tools or any other exogenous reagents. Specifically, by focusing and scanning wavelength-tuned low-power femtosecond laser pulses on the plasma membrane for multiphoton excitation, we directly induced Ca^2+^ influx in cultured cells. Mechanistic study reveals that photoexcited flavins covalently bind cysteine residues in Orai1 via thioether bonds, which facilitates Orai1 polymerization to form store-operated calcium channels (SOCs) independently of STIM1, a protein generally participating in SOC formation, enabling all-optical activation of Ca^2+^ influx and downstream signaling pathways. Moreover, we used femtoSOC to demonstrate direct neural activation both in brain slices in vitro and in intact brains of living mice in vivo in a spatiotemporal-specific manner, indicating potential utility of femtoSOC.

## Introduction

Ca^2+^ channels are essential to cell birth, life, and death.^1,2^ Among Ca^2+^ channels, store-operated calcium channels (SOCs) are of prominent importance in virtually all cells including excitable cells such as neurons and skeletal muscle cells since they maintain cellular Ca^2+^ balance and regulate Ca^2+^ influx from extracellular milieu for various vital physiological functions such as cell development, growth, differentiation, and apoptosis.^3–5^ They also play an important role in the control of gene expression, secretion, and immune response.^6–8^ The last two decades have seen an extensive study of the molecular mechanism of Ca^2+^ influx through SOCs, leading to a number of findings about the structure and function of SOCs.^5,7,9–11^ According to a widely accepted theory, SOCs are plasma membrane ion channels mainly composed of the pore-forming subunit calcium release-activated calcium channel protein 1 (Orai1) and are activated to open in response to the depletion of Ca^2+^ in the lumen of the endoplasmic reticulum (ER) sensed by the ER-localized protein stromal interaction molecule 1 (STIM1), which polymerizes and relocates near the plasma membrane, where it covalently binds Orai1 and triggers its formation to be hexamers for the formation of SOCs.^12–14^

In the last several years, efforts have been made to develop techniques for external activation of SOCs. For example, Kyung et al. have developed an optogenetic tool, called OptoSTIM1 based on a combination of a plant photoreceptor and the STIM1, which can activate Ca^2+^-selective endogenous CRACs (Ca^2+^ release-activated Ca^2+^ channels, i.e., SOCs), with blue light illumination and induce Ca^2+^ influx to quantitatively control intracellular Ca^2+^ levels in zebrafish embryos and human embryonic stem cells.^15^ Another approach is an optogenetic platform developed by He et al., called OptoCRAC, which can control Ca^2+^ influx through the activation of CRACs in non-excitable cells (e.g., T lymphocytes, macrophages, dendritic cells) with near-infrared light illumination, thereby generating repetitive Ca^2+^ oscillations, regulating Ca^2+^-dependent gene expression, and modulating various Ca^2+^-dependent activities in the cells.^16^ Unfortunately, while these optogenetic techniques are effective and powerful, they require time-consuming, but robust introduction of exogenous optogenetic genes that express the photosensitive proteins into biological systems transiently or stably,^17,18^ due to which the strength of the SOC activation varies, depending on their expression level.

In this study, we present a radically different approach to controlling SOCs. Our method (termed femtoSOC) enables direct control of Ca^2+^ channels solely by ultrafast laser without the need for optogenetic tools or any other exogenous reagents, hence alleviating the requirements which would normally be required for the optogenetic activation of SOCs. Specifically, by focusing and scanning wavelength-tuned low-power femtosecond laser pulses on a small region of the plasma membrane for multiphoton excitation, we directly induced Orai1to covalently bind flavin and polymerize for the formation of SOCs independently of STIM1, which would normally participate in the SOC formation, leading to the activation of Ca^2+^ influx and downstream signaling pathways. Through a series of experiments and validation tests, we developed the molecular mechanism of femtoSOC including the mechanism of Orai1hexamer formation in the SOC formation and the role of the mitochondria and reactive oxygen species. Finally, to show the potential utility of femtoSOC, we used it to demonstrate direct in vitro and in vivo neural activation in brain slices and an intact living mouse brain, respectively, in a spatiotemporal-specific manner. It is important to note that femtoSOC is highly simple as it can be demonstrated on any two-photon microscope system in which the required laser illumination power is only a few milliwatts (lower than the typical laser power used in two-photon microscopy). femtoSOC holds promise for various biomedical applications as a much simpler alternative to optogenetic tools.

## Results

### Basic performance and validation of femtoSOC

**Fig. 1:**
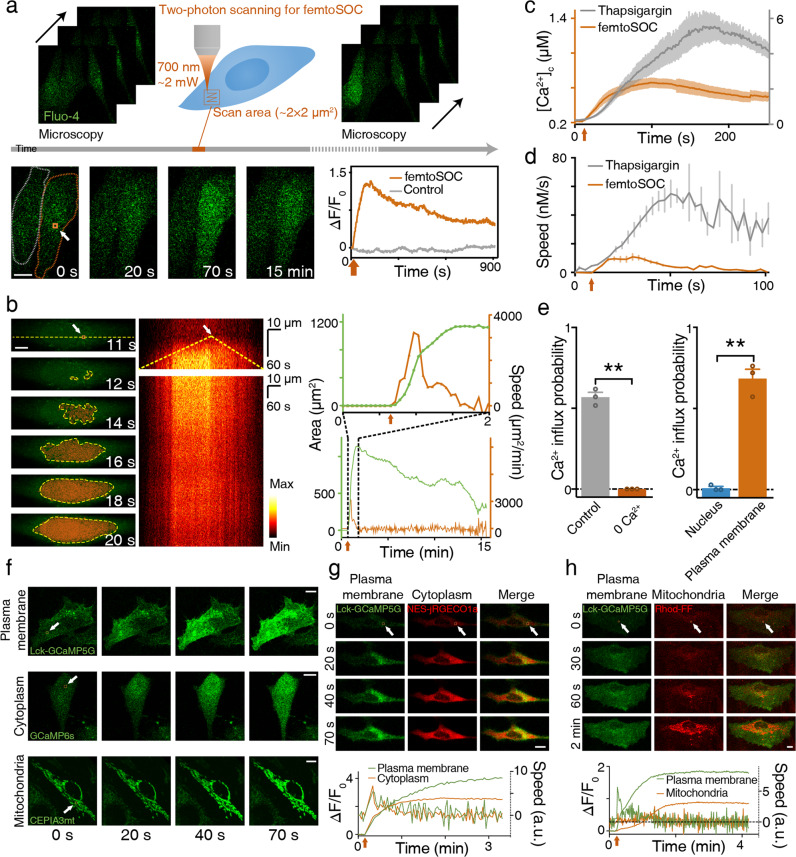
Basic performance of femtoSOC for direct control of SOCs by ultrafast laser. **a** Schematic diagram of femtoSOC. Only a small area (2 × 2 μm^2^) of the plasma membrane is scanned once by the femtosecond laser as a single two-photon microscope frame for 63 ms. The fluorescence in the images indicates the intensity of femtoSOC-induced Ca^2+^ influx (*n* = 50 cells). **b** Diffusion of Ca^2+^ influx from the femtoSOC laser illumination area (*n* = 13 cells). Orange inside the dashed line, the area of fluorescence intensity above a threshold. Right, Ca^2+^ diffusion area and speed. **c** Ca^2+^ level in the cytoplasm (means ± SEM, *n* = 35 cells). **d** Ca^2+^ influx speed (*n* = 35 cells). Gray line, cells treated with TG at 5 μM, showing the Ca^2+^ influx after the Ca^2+^ in ER was depleted as a positive control. Orange line, cells illuminated by femtoSOC. Arrow, the timing of femtoSOC laser illumination. **e** Ca^2+^ influx probability. Left, Ca^2+^-free medium (*n* = 63 cells in 3 independent experiments, *P* = 0.0013). Right, comparison between the nucleus and plasma membrane (*n* = 72 cells in 3 independent experiments, *P* = 0.0067, two-tailed paired *t*-test). **f** Ca^2+^ influx indicated by the localized Ca^2+^-sensitive GFP: Lck-GCaMP5G (plasm membrane), GCaMP6s (cytoplasm), and CEPIA3mt (mitochondria), respectively. **g** Simultaneous visualization of Ca^2+^ influx through the plasma membrane and cytoplasm. **h** Ca^2+^ influx through the plasma membrane and mitochondria. Lower panels, Ca^2+^ level and influx speed in the organelles. Box, femtoSOC laser illumination area. Arrow, femtoSOC laser illumination position. Scale bars, 10 μm.

As schematically shown in Fig. 1a, the basic operation of femtoSOC is to scan a small area of the plasma membrane of a target cell with a femtosecond pulse laser whose power is typical (or even lower) for two-photon microscopy without the need for any special preparation for the cell. Specifically, a multiphoton confocal microscope (A1 MP+, Nikon and SP8, Leica) equipped with a femtosecond pulse laser (1.4–4.0 mW, 700 nm, MaiTai DeepSee, Spectra-Physics) was used without any special modifications for both performing femtoSOC on cells for a duration of 63–500 ms and imaging of the cells before, during, and after femtoSOC. As a proof-of-principle demonstration, we used HeLa cells in culture medium loaded with Fluo-4/AM for Ca^2+^ fluorescent imaging. As shown in Fig. 1a, dramatic Ca^2+^ rise immediately after the femtoSOC laser illumination was evident. As shown in Fig. 1b, the elevated Ca^2+^ concentration was found to start in the femtoSOC laser illumination area and soon diffused to the entire cell in about 10 s, which eventually led to an overall intracellular Ca^2+^ concentration increase from about 200 nM to about 600 nM (Fig. 1c), estimated by a fluorescence quantification method.^19^ The speed of Ca^2+^ concentration increase was found significantly different between the femtoSOC-excited cells and cells treated with thapsigargin (TG), an inhibitor that suppresses the sarco/endoplasmic reticulum Ca^2+^ ATPase, depletes Ca^2+^ in ER, and allows an influx of Ca^2+^ into the cytosol (Fig. 1d; Supplementary information, Fig. S1). The membrane integrity and viability of the femtoSOC-excited cells were not influenced by the laser illumination (Supplementary information, Fig. S2). Furthermore, a series of validation tests verified femtoSOC-induced Ca^2+^ influx. Firstly, to determine if the source of the Ca^2+^ elevation was extracellular medium or intracellular Ca^2+^ stores, we replaced the cell buffer with Ca^2+^-free medium and found that no Ca^2+^ rise was present after the femtoSOC laser illumination (Fig. 1e). Secondly, we also found that no Ca^2+^ rise was present either after localizing the femtosecond-laser focus inside the cell nucleus in normal medium. The photon density at plasma membrane above nucleus, which was out of focus, was too low for multiphoton excitation enabling excitation to nucleus solely. Thirdly, using localized fluorescent Ca^2+^-indicative proteins (Lck-GCaMP5G, GCaMP6s, and CEPIA3mt) transfected into cells (Supplementary information, Fig. S3), we visualized Ca^2+^ dynamics in the plasma membrane, cytoplasm, and mitochondria, respectively and found that the Ca^2+^ diffusion in both the plasma membrane and cytoplasm clearly initiated from the femtoSOC laser illumination area (Fig. 1f). The mitochondria also showed a Ca^2+^ rise, indicating a global Ca^2+^ rise in the cell. The occurrence of the Ca^2+^ influx and diffusion in these organelles was found nearly simultaneous with a small time delay (Fig. 1g, h). This Ca^2+^ diffusion was not significantly influenced by blocking ryanodine receptors (RyR) (Supplementary information, Fig. S4).

### Characterization of femtoSOC

**Fig. 2:**
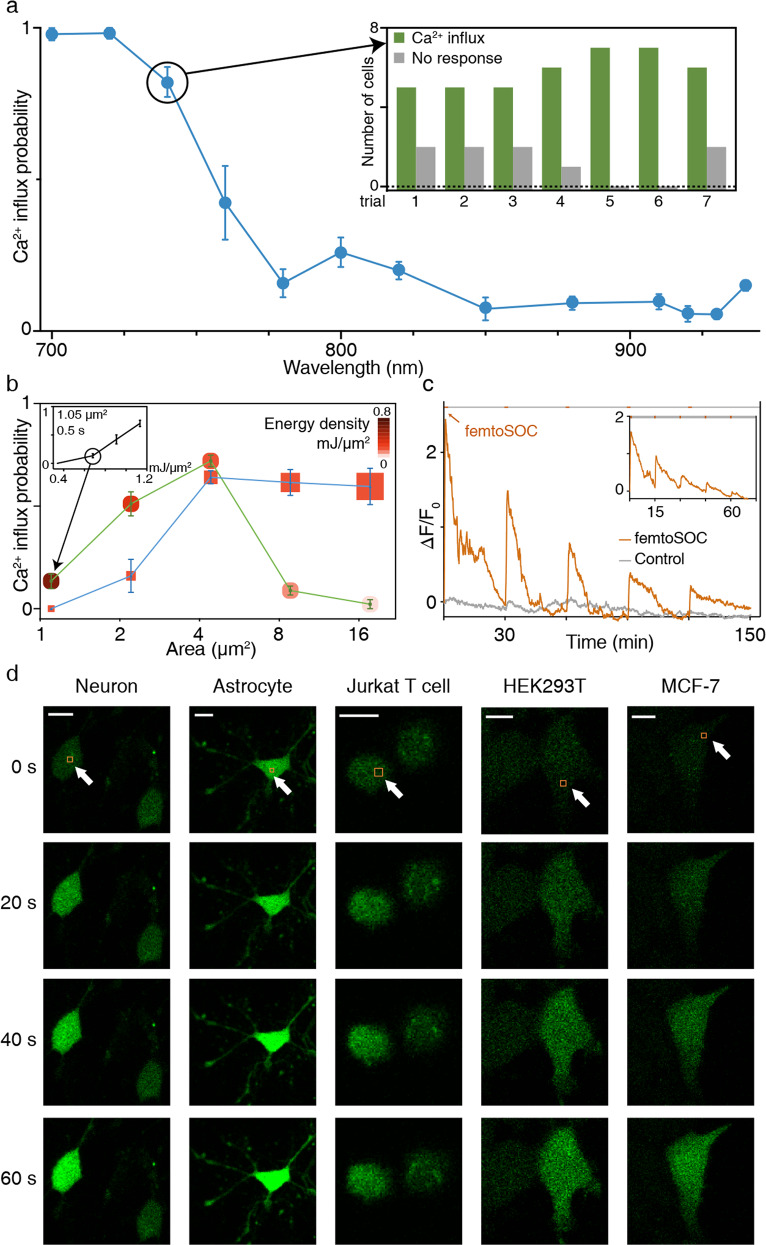
Characterization of femtoSOC. **a** Spectrum of femtoSOC-induced Ca^2+^ influx. Inset, at each wavelength, femtoSOC was applied to 7 independent groups of cells (*n* = 7–20 cells in each group). **b** femtoSOC-induced Ca^2+^ influx probability versus energy density and femtoSOC laser illumination area. Blue, constant energy density at 0.17 mJ/μm^2^. The size of each box indicates the femtoSOC laser illumination duration. Green, constant total energy at 0.68 mJ with the same femtoSOC laser illumination duration (*n* = 15–27 cells in each independent measurement, repeated 3–6 times, at each point). Inset, constant femtoSOC laser illumination area at 1.1 μm^2^ for a duration 0.5 s. **c** femtoSOC-induced Ca^2+^ influx performed multiple times (every 30 min, *n* = 21 cells). The overall decrease of the fluorescence intensity was due to the photobleaching and leakage of Fluo-4 during the long-term observation. Inset, femtoSOC-induced Ca^2+^ influx performed every 15 min. **d** Versatility of femtoSOC. femtoSOC was performed on primary mouse neurons (*n* = 16 cells), mouse astrocytes (*n* = 11 cells), Jurkat T cells (*n* = 62 cells), HEK293T cells (*n* = 62 cells), and MCF-7 cells (*n* = 49 cells). Box, femtoSOC laser illumination area. Arrow, femtoSOC laser illumination position. Scale bars, 10 μm.

To characterize the efficiency of femtoSOC, we varied its conditions such as the laser wavelength, energy density, scan area, scan frequency, and cell line. First, we tuned the laser wavelength from 700 nm to 960 nm with the average laser power and pulse width constant. We measured the probability of Ca^2+^ influx after the femtoSOC laser illumination at each laser wavelength, defined by the ratio of the number of cells with Ca^2+^ influx to the total number of cells illuminated. As shown in Fig. 2a, the probability was found to decrease sharply as the laser wavelength increased, with half the peak value at 750 nm. This narrow bandwidth is presumably due to two-photon absorption of molecules, not physical damage (e.g., photoporation by multiphoton ionization) with little dependence on the laser wavelength.^20^ This spectrum also suggests that photothermal effects contributed little to Ca^2+^ influx. The probability at 960 nm was near zero despite the fact that the water absorption coefficient at 960 nm is > 10 times larger than at 700 nm. To further confirm this, a continuous-wave (CW) laser at 760 nm with 10 times higher power was used to stimulate cells as a control experiment of femtoSOC at the same laser wavelength. No cells showed Ca^2+^ rise in the control experiment (Supplementary information, Fig. S5). Second, we measured the influence of the laser energy and scan area on Ca^2+^ influx by measuring it at various scan areas. As shown in Fig. 2b, when the energy density was kept constant (blue curve), the probability of Ca^2+^ influx increased and remained at the same level as the scan area increased. On the other hand, when the total laser energy was kept constant (green curve), the probability increased, reached a maximum value, and dropped as the scan area increased. The probability was found to be zero at a scan area of 1 μm^2^ although the energy density was quite high (Supplementary information, Fig. S6). Third, we performed a test of multiple femtoSOC scans to observe multiple occurrences of Ca^2+^ influx on the same cell and verified its feasibility as shown in Fig. 2c. Finally, to show femtoSOC’s applicability to various cell types, we performed femtoSOC on primary cultured neurons and astrocytes of mice, Jurkat T cells, HEK293T cells, and MCF-7 cells. As shown in Fig. 2d, all these cells showed obvious femtoSOC-induced Ca^2+^ influx.

### Verification of femtoSOC-induced SOC formation by Orai1

**Fig. 3:**
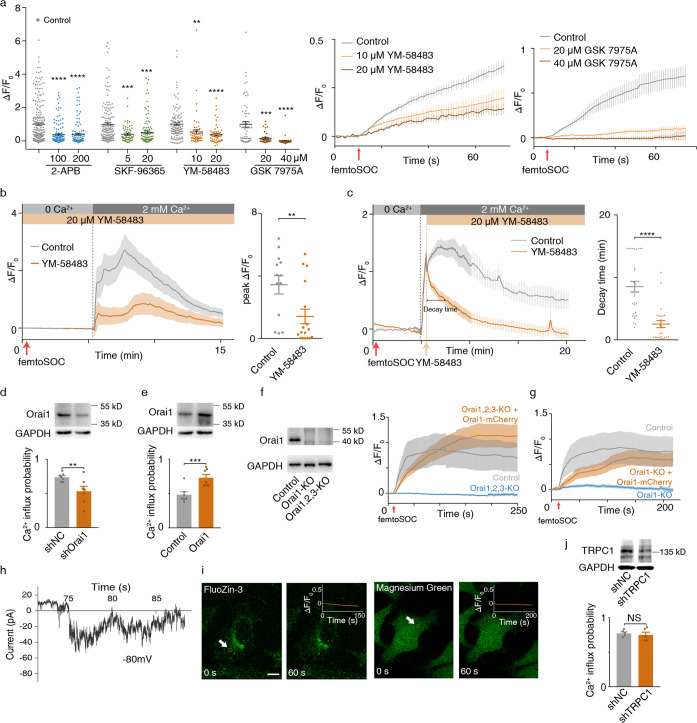
Orai1’s specific participation in femtoSOC-induced SOC formation. **a** Ca^2+^ influx intensity of cells treated with 2-APB (*n* = 132, 4 trials; *n* = 129 cells, 5 trials), SKF-96365 (*n* = 60, 4 trials; *n* = 108 cells, 6 trials), YM-58483 (*n* = 57, 3 trials; *n* = 57 cells, 3 trials), and GSK7975A (*n* = 60 cells, 3 trials; *n* = 60 cells, 3 trials) at two different concentrations for a duration of 70 s after the femtoSOC laser illumination. **b** Add-back test showing Ca^2+^ influx suppressed by adding YM-58483 before the femtoSOC laser illumination. Red arrow, timing of the femtoSOC laser illumination. Right, statistics of the measured Ca^2+^ influx peak level with or without YM-58483 (*n* = 13 cells and *n* = 17 cells in the control and YM-58483 groups, respectively, *P* = 0.0061). **c** Add-back test showing Ca^2+^ influx suppressed by adding YM-58483 30 s after the femtoSOC laser illumination. Red arrow, timing of the femtoSOC laser illumination. Orange arrow, timing of the YM-58483 addition. Right, statistics of the Ca^2+^ influx decay time defined by the duration of the decay to reach the half maximum of the peak value (*n* = 30 cells and *n* = 24 cells in the control and YM-58483 groups, respectively, *P* < 0.0001). **d** Ca^2+^ influx probability of cells with Orai1 knockdown (*n* = 144 cells, *P* = 0.0057). Inset, western blot analysis of Orai1 knockdown. **e** Ca^2+^ influx probability of cells with Orai1 overexpression (*n* = 135 cells, *P* = 0.001). Inset, western blot analysis of Orai1 overexpression. **f** Ca^2+^ influx intensity in Orai1/2/3-KO cells compared with that in control cells or that in Orai1/2/3-KO cells with Orai1-mCherry introduced. **g** No Ca^2+^ influx in Orai1-KO cells. **h** Electrophysiological current of Ca^2+^ influx measured by the patch-clamp technique. **i** Confirmed absence of Zn^2+^ and Mg^2+^ influx. **j** Ca^2+^ influx probability of cells with TRPC1 knockdown by shRNA (*n* = 114 cells, 5 trials, *P* = 0.3369). Inset, western blot analysis of TRPC1 knockdown. *P < 0.05. **P < 0.01. ***P < 0.001. ****P < 0.0001. NS, no significant difference. Scale bar, 10 μm.

To verify if femtoSOC-induced Ca^2+^ influx is through SOCs, we performed a series of validation tests. First, we used various blockers of Ca^2+^ channels: two broad-spectrum inhibitors, namely (i) 2-aminoethoxydiphenyl borate (2-APB) which is an inhibitor of transient receptor potential (TRP) channels and SOCs, (ii) SKF-96365, which is a blocker of voltage-gated Ca^2+^ channels, transient receptor potential cation (TRPC) channels, and SOCs; and two specific blockers of SOCs, namely (iii) YM-58483 and (iv) GSK-7975A. As shown in Fig. 3a, both 2-APB and SKF-96365 showed inhibition effects on Ca^2+^ influx after the femtoSOC laser illumination. Also, YM-58483 and GSK-7975A strongly suppressed Ca^2+^ influx after the femtoSOC laser illumination (Fig. 3a). It is important to note that GSK-7975A at 40 μM was able to totally inhibit the probability of Ca^2+^ influx to zero. These results firmly confirm that femtoSOC-induced Ca^2+^ influx is through SOCs. Second, we performed a classic Ca^2+^ add-back test to verify if SOCs are opened by femtoSOC. We incubated cells in Ca^2+^-free medium in the presence of YM-58483 and observed no Ca^2+^ influx after the femtoSOC laser illumination (Fig. 3b). After a 5-min rest, we added Ca^2+^ into the medium with a final Ca^2+^ concentration of 2 mM. A significant level of Ca^2+^ influx was identified in the control experiment (i.e., without YM-58483) in contrast to a slight level of Ca^2+^ influx observed in the presence of YM-58483. These results indicate that SOCs were opened by femtoSOC. Third, we performed a similar test to verify if Ca^2+^ influx is solely through SOCs. Specifically, in the Ca^2+^ add-back test, YM-58483 was added into Ca^2+^-free medium 30 s after adding Ca^2+^ back. As shown in Fig. 3c, Ca^2+^ influx was found to have already occurred for 30 s, but it was immediately and significantly suppressed by adding YM-58483 to the medium. Moreover, the decay time of the Ca^2+^ level was found significantly shorter than that in the control. Therefore, these results show that Ca^2+^ entered cells solely through SOCs.

To verify if Orail1 directly participates in the femtoSOC-induced formation of SOCs, we conducted a series of experiments. First, we used cells with Orai1 knockdown and overexpression and identified decreased and increased Ca^2+^ influx probability values in the cell groups as shown in Fig. 3d, e, respectively. Second, to confirm the role of Orai1, we used cells with CRISPR-based Orai1/2/3 knockout and Orai1 knockout (Orai1/2/3-KO and Orai1-KO, respectively). Immediately, we found no Ca^2+^ influx in the Orai1/2/3-KO cells after the femtoSOC (Fig. 3f). In the rescue test, by transfecting Orai1-mCherry into the Orai1/2/3-KO cells, only Orai1 existed in the cells but no Orai2 or Orai3 there. We then performed femtoSOC illumination and found recovered Ca^2+^ influx (Fig. 3f), suggesting the essential role of Orai1. Furthermore, if only Orai1 was knockout, as shown in Fig. 3g, no Ca^2+^ influx after the femtoSOC treatment was observed either, indicating Orai2 and Orai3 did not participate in the femtoSOC activation of SOC channels. As a rescue test, when the Orai1-KO cells were transfected back with Orai1-mCherry, we identified the recovery of Ca^2+^ influx. This further confirms the validity of this experiment. Therefore, these results show that femtoSOC-induced Ca^2+^ influx is through SOCs formed by Orai1. Third, we used the patch-clamp technique to measure the electrophysiological current of Ca^2+^ influx and found it to be at the level of 5–10 pA for a duration of > 10 s after the femtoSOC laser illumination, which is consistent with the typical current value reported in literature^5,14^ (Fig. 3h). Fourth, we investigated the specificity of femtoSOC-induced Ca^2+^ influx by using other cations such as Zn^2+^ and Mg^2+^, which can be detected with FluoZin-3/AM and MgGreen, respectively. Specifically, we incubated cells in the presence of Zn^2+^ (1–7 μM) and Mg^2+^ (1–5 mM) in culture medium separately and observed no fluorescence increase in both cell groups after the femtoSOC laser illumination as shown in Fig. 3i (very different from the positive control shown in Supplementary information, Fig. S8), indicating that femtoSOC does not induce any influx of Mg^2+^ and Zn^2+^. Finally, we investigated if femtoSOC also activates transient receptor potential channels (TRPCs), a group of non-specific cation channels. Specifically, we used cells with TRPC1 knockdown and observed very little change in Ca^2+^ influx probability after the femtoSOC laser illumination as shown in Fig. 3j, indicating that TRPC1s are not activated by femtoSOC.

### Molecular mechanism of femtoSOC

**Fig. 4:**
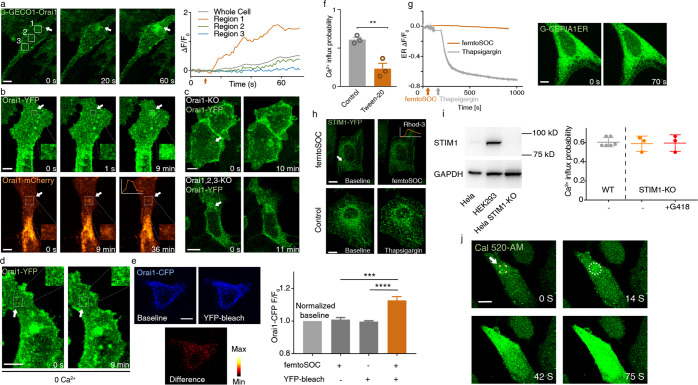
Molecular mechanism of femtoSOC. **a** Ca^2+^ influx through Orai1 indicated by G-GECO1-Orai1 (*n* = 24 cells). White boxes, three regions of interest. Right, Ca^2+^ influx intensity in those regions. **b** Orai1 aggregation in the femtoSOC laser illumination area (orange box) indicated by Orai1-YFP (upper) and Orai1-mCherry (lower) with the illumination at 0 s (*n* = 65 cells). **c** Orai1 aggregation in Orai1-KO cells and Orai1/2/3-KO cells indicated by Orai1-YFP (*n* = 16 cells). **d** Orai1 aggregation in Ca^2+^-free medium indicated by Orai1-YFP (*n* = 17 cells). **e** FRET microscopy of cells simultaneously indicated by Orai1-CFP and Orai1-YFP. **f** Ca^2+^ influx probability in the presence of Tween-20 (0.025%, v/v, *n* = 28 cells in 3 trials). **g** Ca^2+^ store in the ER, indicated by G-CEPIA1ER (*n* = 39 cells). **h** Absence of STIM1-YFP aggregation in cells (left, *n* = 27 cells). Inset, Ca^2+^ influx indicated by Rhod-3. STIM1-YFP puncta or clusters in the positive control after TG (5 μM) treatment (right, *n* = 19 cells). **i** Left, western blot analysis of STIM1. Right, Ca^2+^ influx probability of STIM1-KO cells with (*n* = 90 cells) and without G418 incubation (*n* = 90 cells) in comparison with that of wild-type cells (WT, *n* = 162 cells). **j** The Ca^2+^ diffusion in the STIM1-KO cells after femtoSOC in the Ca^2+^ add-back test. **P < 0.01. ***P = 0.0002. ****P < 0.0001. Scale bars, 10 μm.

To study the molecular mechanism of femtoSOC, we conducted a series of experiments. First, we investigated the dynamic change of Orai1 associated with Ca^2+^ influx after the femtoSOC laser illumination. Specifically, we used plasmid G-GECO1-Orai1, a fusion of the Ca^2+^-sensitive green fluorescent protein (GFP) with Orai1 that expressed in HeLa cells to simultaneously visualize both Orai1 activity and Ca^2+^ influx. As shown in Fig. 4a, Ca^2+^ influx occurred only in the two-photon scanning area where Orai1 also showed its aggregation at the very beginning after femtoSOC, indicating that Orai1 formed SOCs in the area. Second, to confirm Orai1 aggregation during the femtoSOC laser illumination, we monitored Orai1’s dynamic location with Orai1-YFP and Orai1-mCherry. Figure 4b shows obvious Orai1 aggregation in the two-photon scan area which diffused away after a short time. Again, using Orai1-KO and Orai1/2/3-KO cells transferred back with Orai1-YFP, we identified the presence of Orai1-YFP aggregation in the cells as shown in Fig. 4c, further supporting femtoSOC-induced Orai1 aggregation. To exclude the possibility that Orai1 aggregation was induced by Ca^2+^ influx, we monitored Ca^2+^ influx in cells cultured in Ca^2+^-free medium and found no Ca^2+^ influx under this condition while Orai1-YFP aggregation was present as shown in Fig. 4d. Third, to verify if Orai1 formed hexamers for the formation of SOCs during its aggregation process, we measured the distance between Orai1 molecules after the femtoSOC laser illumination by Förster resonance energy transfer (FRET) microscopy analysis of cells simultaneously transfected with Orai1-CFP and Orai1-YFP. Our investigation was based on the hypothesis that if Orai1 polymerizes after the femtoSOC laser illumination, Orai1-CFP’s fluorescence will transfer to Orai1-YFP by FRET, but if Orai1-YFP is photobleached, the CFP fluorescence energy cannot be transferred to the YFP, such that Orai1-CFP’s fluorescence should be higher than that in the above case. In this regard, we randomly selected cells after the femtoSOC laser illumination and bleach Orai1-YFP by intense 488-nm laser scanning for 3 s. As shown in Fig. 4e, Orai1-CFP’s fluorescence significantly increased in the photobleached cell group while no CFP fluorescence increase was present in the control group without YFP photobleaching or the group without the femtoSOC laser illumination. These results support Orai1’s polymerization in femtoSOC. Fourth, to verify that Orai1’s polymerization is caused by its hydrophobic interaction,^21,22^ we used Tween-20 to disturb the formation of hydrophobic bonds and hence suppress Orai1’s polymerization. As shown in Fig. 4f, the femtoSOC-induced Ca^2+^ influx probability significantly decreased in the presence of Tween-20 (0.025% v/v), further supporting that Orai1 molecules polymerize via hydrophobic bonds.

Finally, we studied the potential role of STIM1 in the formation of Orai1 hexamers by measuring the localized Ca^2+^ level in the ER during the femtoSOC laser illumination. The localized Ca^2+^ level in the ER was fluorescently indicated by G-CEPIA1ER. After the femtoSOC laser illumination, no fluorescence decrease was observed as shown in Fig. 4g, indicating that the femtoSOC laser illumination did not induce any Ca^2+^ release from the ER. Next, we observed STIM1’s dynamics, which, according to the classical SOC regulation mechanism, polymerizes if Ca^2+^ is depleted in the ER, migrates to the cytoplasm–membrane–ER junction, and interacts with Orai1 to form SOCs^9,23,24^ as shown in Fig. 4h (control), but on the contrary, we observed no STIM1-YFP puncta or clusters during or after the femtoSOC laser illumination as shown in Fig. 4h, which is consistent with the above results that the femtoSOC laser illumination did not influence the Ca^2+^ store in the ER. Therefore, STIM1 did not participate in the SOC formation by Orai1. To further confirm these results, we used STIM1-KO HeLa cells obtained by CRISPR and measured their Ca^2+^ influx probability. As shown in Fig. 4i, these cells showed no change in the probability. Furthermore, we measured the Ca^2+^ influx probability of STIM1-KO HeLa cells incubated in medium with a specific inhibitor of STIM2, G418, and found no change in the Ca^2+^ influx probability either. Moreover, we performed femtoSOC in STIM1-KO cells in Ca^2+^-free medium. After 5-s rest, 200 μM Ca^2+^ was added back into the medium. Immediately Ca^2+^ influx could be observed in the femtoSOC area. At the first 10 s, the Ca^2+^ influx stayed in the illuminated region and soon diffused out to the whole cell (Fig. 4j), indicating the Ca^2+^ influx through femtoSOC-activated SOC independent of STIM1. These results show that STIM1 does not participate in femtoSOC-induced Ca^2+^ influx.

### Molecular mechanism of Orai1 hexamer formation in femtoSOC

**Fig. 5:**
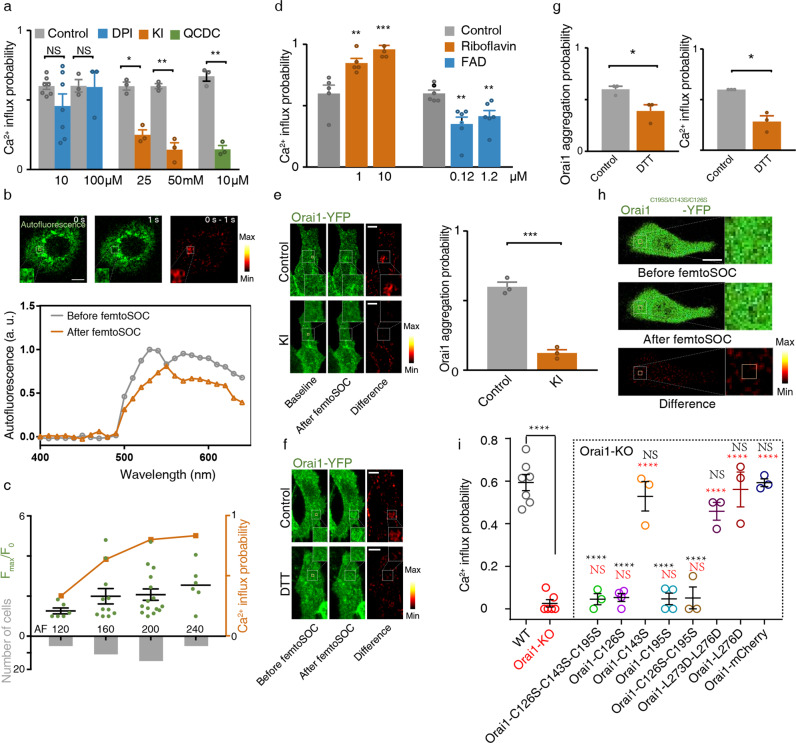
Molecular mechanism of Orai1 hexamer formation in femtoSOC. **a** Ca^2+^ influx probability of cells incubated with DPI (*n* = 81 cells and 3 trials for both groups, *P* = 0.0154 and *P* = 0.0024 for 10 µM and 100 µM DPI, respectively) and KI (*n* = 144 cells, 7 trials, *P* = 0.007 for 25 mM KI; *n* = 63 cells, 3 trials, *P* = 0.4636 for 50 mM KI). **b** Autofluorescence images and typical spectrum (*n* = 30 cells) of flavin before and after femtoSOC, with their difference shown in the heat map (*n* = 10 cells). Box, femtoSOC laser illumination area. **c** Ca^2+^ influx probability of cells with various flavin concentrations. **d** Ca^2+^ influx probability of cells incubated with Riboflavin (*n* = 150 cells, 5 trials for both) and FAD (*n* = 144 cells, 6 trials for 0.12 µM; *n* = 132 cells, 6 trials for 1.2 µM). **e** Aggregation probability of Orai1 suppressed by KI (*n* = 27 cells). **f** Aggregation probability of Orai1 suppressed by DTT (*n* = 27 cells). **g** Aggregation probability of Orai1 and Ca^2+^ influx probability of cells suppressed by DTT (*n* = 31 cells). **h** Absence of Orai1 aggregation in Orai1^C195S-C143S-C126S^-YFP-expressing cells. **i** Ca^2+^ influx probability of cells expressing Orai1^C195S-C143S-C126S^, Orai1^C126S^, Orai1^C195S^, Orai1^C126S-C195S^, Orai1^C143S^, Orai1^L273D-L276D^, and Orai1^L276D^ cells. *P < 0.05. **P < 0.01. ***P < 0.001. ****P < 0.0001. NS, no significant difference. Black stars, comparison with the WT group. Red stars, comparison with the Orai1-KO group. Scale bars, 10 μm.

To further investigate the unique molecular mechanism of femtoSOC which is very different from the classical mechanism of STIM1-triggered Ca^2+^ influx, we conducted a series of experiments with regard to Orai1 aggregation and polymerization in femtoSOC. Specifically, in light of a close similarity between the probability spectrum of femtoSOC-induced Ca^2+^ influx and the two-photon absorption spectrum of flavin, we hypothesized on flavin’s contribution to Orai1 polymerization. First, we incubated cells with potassium iodide (KI) and quinacrine dihydrochloride (QCDC), which are quenchers/inhibitors of photoexcited flavin, and diphenyleneiodonium (DPI), an inhibitor of protein-bound flavin, and measured the femtoSOC-induced Ca^2+^ influx probability in all groups. As shown in Fig. 5a, the KI and QCDC group showed a significantly-decreased probability value while the DPI group showed no change. Here, the KI treatment itself did not exhibit any influence on Ca^2+^ influx through SOCs (Supplementary information, Fig. S9). These results indicate that photoexcited flavin by the femtosecond laser is necessary for femtoSOC. We further verified that the free flavin participates in the femtoSOC excitation by autofluorescence measurement. As shown in Fig. 5b, autofluorescence from flavin in the femtoSOC laser illumination area (excited by a 488-nm laser) significantly changed after the femtoSOC laser illumination, indicating that flavin in the area is excited so that the autofluorescence spectrum varies. Second, we used flavin’s autofluorescence from the entire cell to study the intracellular flavin content. As shown in Fig. 5c, both the Ca^2+^ influx intensity and probability of cells were found higher in cells with higher autofluoresence level of flavin despite it is highly heterogeneous, confirming that a higher flavin concentration leads to a higher femtoSOC efficiency. To further verify this, we regulated the cellular flavin concentration by adding riboflavin (RF)^25^ to the cell medium for one-day culture. As shown in Fig. 5d, the Ca^2+^ influx probability of these cells increased significantly, while that of cells cultured with flavin adenine dinucleotide (FAD) which suppressed the cellular uptake of RF^26^ decreased significantly. Taken all data above, flavin is necessary for femtoSOC-induced Ca^2+^ influx.

We investigated how flavin participates in the formation of SOCs in the process of femtoSOC. As shown in Fig. 5e, Orai1 aggregation was significantly inhibited in the presence of KI that quenched photoexcited flavins. Furthermore, based on the assumption that femtoSOC-excited flavins form thioether bonds with cysteine residues in Orai1,^27,28^ we treated cells with dithiothreitol (DTT) that could protect the –SH group of cysteine in Orai1 from forming thioether bonds with flavins. The DTT treatment itself did not influence the function of SOC gating (Supplementary information, Fig. S10). As shown in Fig. 5f and g, the femtoSOC-induced aggregation of Orai1 and the Ca^2+^ influx probability of the cells were significantly suppressed in the presence of DTT. These results suggest that femtoSOC-excited flavins covalently interact with the cysteine residues in Orai1 via thioether bonds.

To screen the cysteine residues of Orai1 involved in the thioether bond formation, we mutated three intrinsic cysteine to serine to inhibit any thioether bond formation between them.^29^ The Orai1 triple mutant (C195S-C143S-C126S) was genetically labeled with YFP and transfected into cells. As shown in Fig. 5h, no aggregation of Orai1 was found when all three cysteine residues were mutated, further confirming flavin interacts with Cys in Orai1 via the thioether bond. Then we transfected Orai1 mutants, namely Orai1^C126S^, Orai1^C143S^, Orai1^C195S^, Orai1^C126S-C195S^ and Orai1^C195S-C143S-C126S^, into Orai1-KO cells. Ca^2+^ influx could occur normally when treated with TG (Supplementary information, Fig. S11). After femtoSOC excitation, little Ca^2+^ influx was found in Orai1-KO or Orai1^C195S-C143S-C126S^ cells (Fig. 5i). The Ca^2+^ influx probability of Orai1^C143S^ cells was close to that of the wild-type cells, but in Orai1^C126S^ and Orai1^C195S^ cells, nearly no Ca^2+^ influx was observed after femtoSOC. The Ca^2+^ influx probability of Orai1^C126S-C195S^ cells further verified this observation. Therefore, Cys126 and Cys195 of Orai1 are responsible for bonding with photoexcited flavins. Finally, to test if Leu273 and Leu276 in Orai1 contribute to hydrophobic interaction in the formation of Orai1 hexamer induced by femtoSOC, Orai1-KO cells were transfected with Orai1^L273D-L276D^ and Orai1^L276D^ mutants, respectively, and then tested by femtoSOC. No significant difference in Ca^2+^ influx probability was found in mutant groups compared with the WT group. Therefore, femtoSOC-excited flavin covalently binds Orai1 via Cys126 and Cys195 simultaneously.

### Verification of the non-participation of the mitochondria and reactive oxygen species in femtoSOC

**Fig. 6:**
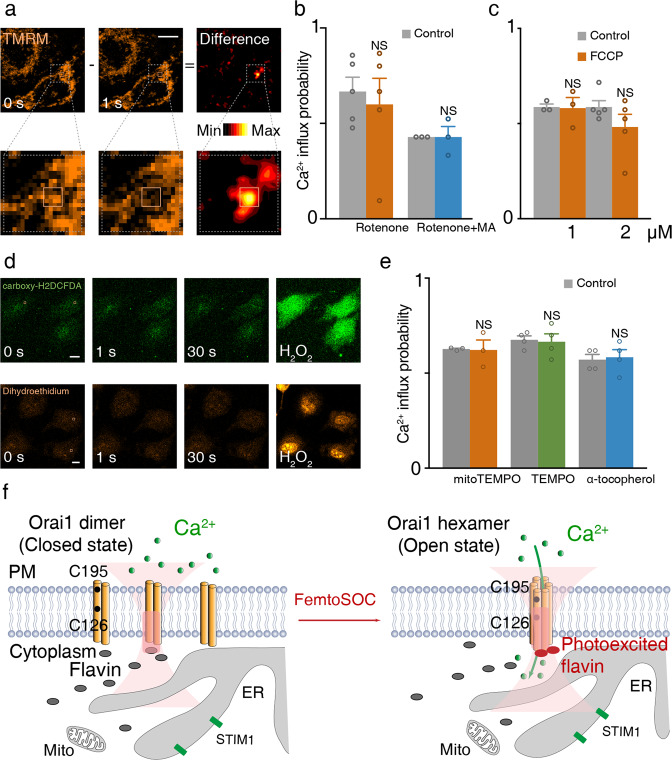
Non-participation of the mitochondria and reactive oxygen species in femtoSOC. **a** MMP before and after the femtoSOC laser illumination. The difference between them is also shown (*n* = 30 cells). **b** Ca^2+^ influx probability of cells in the presence of rotenone (*n* = 93 cells, 5 independent experiments) and both rotenone and MA (*n* = 63 cells, 3 independent experiments). **c** Ca^2+^ influx probability of cells after FCCP treatment (*n* = 72 cells, 3 independent experiments) in comparison with that of cells in the control group (*n* = 108 cells, 5 independent experiments). **d** Absence of ROS activity indicated by DCF and DHE in comparison with the positive control (right, 30 mM H_2_O_2_ treatment). Boxes, femtoSOC laser illumination areas. **e** Ca^2+^ influx probability of cells in the presence of ROS scavengers (*n* = 72 cells, 3 trials for 20 μM mitoTEMPO; *n* = 102 cells, 4 trials for 200 μM TEMPO; *n* = 84 cells, 4 trials for 100 μM α-tocopherol). **f** Schematic diagram of the general mechanism of femtoSOC. NS, no significant difference. Scale bars, 10 μm.

Considering that flavin is involved in intracellular redox reactions and is a vital component of mitochondrial energy production, we investigated a potential influence of femtoSOC on the mitochondria. As shown in Fig. 6a, the mitochondrial membrane potential (MMP) of the entire cell incubated in tetramethylrhodamine, methyl ester (TMRM) maintained a constant level outside of the femtoSOC laser illumination area. Next, we investigated if the mitochondrial respiration process is related to femtoSOC-induced Ca^2+^ influx. Rotenone was used to inhibit the electron transport chain (ETC) at complex I. Malonic acid (MA), an inhibitor of ETC at complex II, was also added to the cell medium along with rotenone. As shown in Fig. 6b, the femtoSOC-induced Ca^2+^ influx probability was not influenced in the presence of rotenone or both rotenone and MA. Finally, cells were treated with FCCP, a potent mitochondrial oxidative phosphorylation uncoupler, to terminate the mitochondrial respiration and depolarize the MMP, but the femtoSOC-induced Ca^2+^ influx probability was also not influenced as shown in Fig. 6c. These results indicate that the femtoSOC is not influenced by mitochondria.

Furthermore, we studied a potential influence of femtoSOC on the reactive oxygen species (ROS). Figure 6d shows that no ROS rise was present after the femtoSOC laser illumination as indicated by carboxy-H_2_DCFDA (DCF) or dihydroethidium (DHE). Also, as shown in Fig. 6e and Supplementary information, Fig. S12, in the presence of mitoTEMPO (20 μM, a mitochondria-targeted ROS scavenger), TEMPO (200 μM and 1 mM, a general cellular ROS scavenger), and α-tocopherol (α-TOC, 100 μM, a lipid-soluble antioxidant that prevents free peroxidation/hydroperoxyl radicals, singlet oxygen, and oxidative damage to the plasma membrane), the femtoSOC-induced Ca^2+^ influx probability was not influenced by any of the ROS scavengers. These results show that the ROS is not related to or influenced by femtoSOC. Taken together, we summarize the total femtoSOC process in Fig. 6f.

### Verification of femtoSOC-induced activation of downstream signaling pathways

**Fig. 7:**
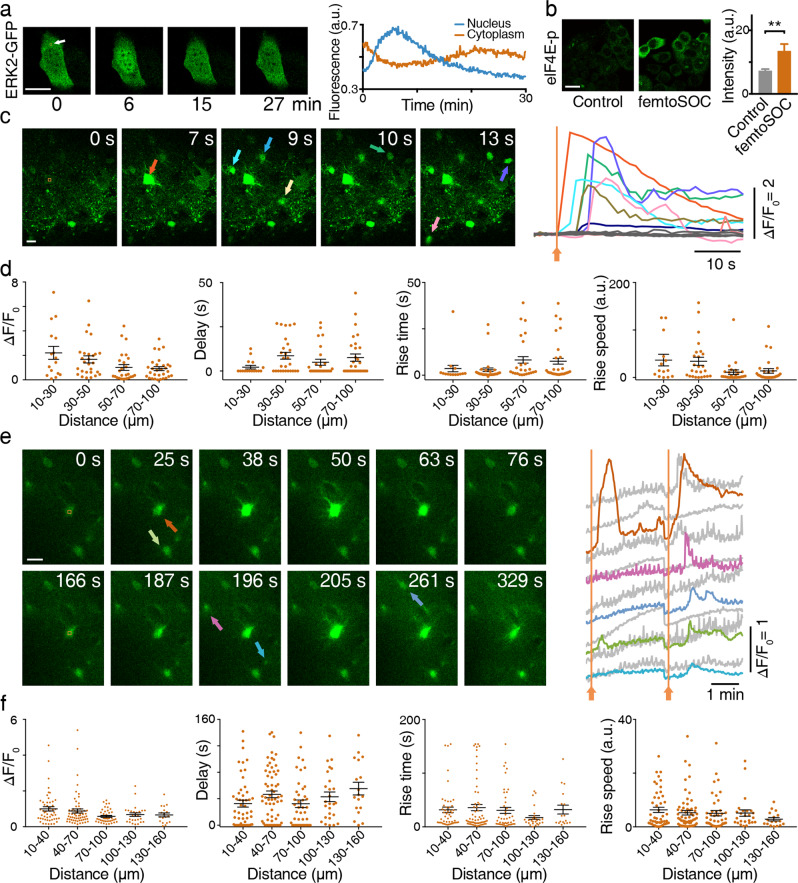
FemtoSOC-induced activation of the ERK and neurons in vitro and in vivo. **a** Translocation of ERK from the cytoplasm to the nucleus and from the nucleus to the cytoplasm, indicated by ERK2-GFP (*n* = 50 cells). Arrow, femtoSOC laser illumination location. **b** Upregulation of eIF4E-p after femtoSOC, indicated by immunofluorescence microscopy (*n* = 16 cells) in comparison with the control (*n* = 18 cells). **c** femtoSOC-induced activation of a randomly selected neuron in the hippocampus of a fresh mouse brain slice (indicated by the orange arrow) followed by the response of the neighboring neurons (indicated by arrows in other colors) due to their connections with the activated neuron (*n* = 33 cells in 3 different slices). Right, Ca^2+^ signal levels of the neurons in the field of view. **d** Statistics of the Ca^2+^ influx intensity, the delay between the femtoSOC laser illumination and the Ca^2+^ influx start time, the Ca^2+^ signal rise time, and Ca^2+^ rise speed in the neighboring neurons at different distances from the femtoSOC-activated neuron. **e** Two-time femtoSOC-induced activation of a randomly selected neuron located at about 175 μm depth in an intact living mouse brain at 12 s and 172 s (*n* = 30 neurons from 3 mice). Right, Ca^2+^ signal levels of the neurons (indicated by the arrows with corresponding colors) and other neurons (indicated by gray) in the field of view in response to femtoSOC laser illumination. **f** Statistics of the Ca^2+^ influx intensity, the delay between the femtoSOC laser illumination and the Ca^2+^ influx start time, the Ca^2+^ signal rise time, and Ca^2+^ rise speed in the neighboring neurons at different distances from the femtoSOC-activated neuron. Scale bars, 10 μm.

Since Ca^2+^ influx typically activates intracellular signaling pathways, we investigated femtoSOC-induced activation of extracellular signal-regulated kinases (ERKs), one of the most essential signaling pathways that are involved in the regulation of meiosis, mitosis, and postmitotic functions. Specifically, we transfected the plasmid ERK2-GFP fusion protein into HeLa cells to visualize ERK dynamics in the cytoplasm. As shown in Fig. 7a, the ERK2-GFP migrated into the nucleus after the femtoSOC laser illumination, indicating phosphorylation of the ERK. After about 30 min, the ERK exited the nucleus to the cytoplasm. To further confirm femtoSOC-induced activation of the ERK, we investigated if eIF4E (eukaryotic translation initiation factor 4E), a downstream protein of the ERK pathway, was phosphorylated 24 h after the femtoSOC laser illumination, using HeLa cells (without the ERK2-GFP transfection) cultured for 24 h. As shown in Fig. 7b, the upregulation of phosphorylated eIF4E (eIF4E-p) was found in the cells after the femtoSOC laser illumination by immunofluorescence microscopy. These results show that femtoSOC activates the ERK pathway.

### In vivo neural activation in a mouse brain by femtoSOC

To show the utility of femtoSOC, we used it to demonstrate neural activation in a fresh brain slice and an intact living mouse brain in vivo. Specifically, we demonstrated the activation of any target neuron in a fresh brain slice of normal mice (C57BL/6) within the microscope’s field of view without the need for optogenetics. The brain slice (hippocampus, 300-μm thick) was labeled with Ca^2+^ indicator Cal-520/AM (6 μM), incubated in a perfusion chamber, and observed by a two-photon confocal microscope (SP8, Leica). Neurons at about 150-μm depth in the hippocampus were randomly selected for femtoSOC excitation. After the femtoSOC laser illumination, the femtoSOC-excited neuron immediately exhibited high Ca^2+^ influx (*n* = 34 neurons from 3 brain slices), followed by Ca^2+^ rise in the neighboring neurons as shown in Fig. 7c and Supplementary information, Video S1. The Ca^2+^ signal amplitude and response time in the neighboring neurons were related with the distance from the femtoSOC-excited neuron as shown in Fig. 7d, exhibiting the propagation of Ca^2+^ waves. Furthermore, we demonstrated the activation of neurons in an intact living mouse brain. Neurons in the brain were fluorescently labeled by stereotaxic injection of Cal-520/AM after anesthesia for in vivo observation. The brain was imaged by the same two-photon microscope (SP8, Leica). Target neurons at about 175 μm depth were randomly selected. As shown in Fig. 7e and Supplementary information, Video S2, the target and neighboring neurons showed high Ca^2+^ rise and oscillations after the femtoSOC laser illumination. Two minutes later, a second femtoSOC excitation of the same neuron activated the second Ca^2+^ response in the neurons. As shown in Fig. 7f, the response time, signal amplitude, and rise speed in the neighboring neurons were also related with the distance from the femtoSOC-excited neuron. These results firmly show the utility of femtoSOC to trigger optogenetics-free excitation of neurons in vivo.

## Discussion

It is important to note that the illumination power of femtoSOC is as low as 1–4 mW, lower than the typical laser power used in two-photon microscopy. Also, this is much lower than the femtosecond laser power used for two-photon excitation of optogenetic proteins (20–150 mW). The Ca^2+^ influx spectrum shown in Fig. 2a and the Ca^2+^ influx probability shown in Fig. 2b and Supplementary information, Fig. S6 excluded the contribution of photothermal effects on femtoSOC-induced Ca^2+^ influx. The low laser power, high dependence of femtoSOC on the laser wavelength, and high cell viability (membrane integrity) indicate that femtoSOC-induced photoporation by breakdown or ionization is not present and hence does not contribute to Ca^2+^ influx. Nevertheless, there might still be other possible mechanisms underlying femtoSOC-induced Ca^2+^ influx. Other potential methods of flavin excitation are also worth investigation. For example, blue light might activate flavin and further SOC channels under certain conditions. The photoexcited flavins covalently bind Cys126 in TM2 and Cys195 in TM3 of Orai1 via thioether bonds, respectively, to create a hydrophobic core. The Orai1 molecules might polymerize by hydrophobic bonds to form the hexameric SOC channels.

Orai1 plays a dominant role in femtoSOC since SOCs are mainly composed of Orai1 and STIM1 although there are other Orai family units such as Orai2 and Orai3. Orai3 usually works as an exceptional Orai^30–32^ that does not work for the formation of SOCs under normal conditions, but when SOCs are inhibited, Orai3 may form SOCs to induce very small Ca^2+^ influx. In the presence of Orai1, the contribution of Orai2 to SOCs is mostly suppressed.^31^ Previous reports show that even if Orai2 is knocked down by siRNA, Ca^2+^ influx through SOCs is totally unaffected.^33,34^ Moreover, Orai2 in cells without Orai1 provides very small Ca^2+^ influx.^35^ Recently, it has been found that Orai2 expression in some types of Orai1-KO cells is significantly upregulated,^36^ which accounts for very small Ca^2+^ influx in Orai1-KO cells. Figure 3e, f shows Orai1 is a primary contributor to femtoSOC-induced Ca^2+^ influx. The expression pattern of Orai1 in different cell lines varies a lot. As shown in Supplementary information, Fig. S13, the Orai1 level in MCF-7 is much higher than it in HeLa cells. According to our tests and data shown in Fig. 2d, femtoSOC presents a wide applicability in those different cell types.

The general drawback of femtoSOC comes from the two-photon excitation that requires tight focusing of femtosecond laser. Usually that needs microscope systems instead of direct illumination by laser or even LED. Therefore, it is not easy to simultaneously activate a large amount of cells or neurons of free-running mice. The drawback can be overwhelmed by combining other optical technologies like spatial light modulation for automatic multi-focusing and implantable miniaturized objectives.

femtoSOC can account for the previously reported phenomenon of occasional Ca^2+^ bursts in neurons in two-photon microscopy of mouse brains with an unknown mechanism.^37,38^ More importantly, as shown in our experimental demonstration, femtoSOC has potential to enable optogenetics-free single-neuron activation for neuroscience research and is fully compatible with two-photon microscopy systems used in previous optogenetics research. For example, Tank Group and Hausser Group have reported a two-photon microscope with two femtosecond lasers at different wavelengths for simultaneous two-photon microscopy analysis of neurons and two-photon excitation of neural Ca^2+^ signals by optogenetics.^39,40^ Recently, Harvey Group has found the feature-specific competition of neurons in the V1 (vision) area in mouse brains by using single-neuron optogenetic perturbation.^41^ Deisseroth group and Yuste Group have demonstrated that the activation of several target neurons is sufficient for triggering perception^42^ and controlling the specific behavior of mice,^43^ respectively, by two-photon optogenetic excitation. In these reports, single neurons were stimulated one by one by using two-photon excitation of optogenetic proteins. femtoSOC is expected to be employed in conjunction with the existing two-photon optogenetic excitation systems or significantly simplify them by virtue of no need for introducing exogenous optogenetic genes into biological systems. Such single-cell activation guarantees the cell specificity in this way.

## Materials and Methods

### Fluorescence detection and imaging

For Ca^2+^ imaging, cells were incubated with 1.5 μM Fluo-4/AM (F14201, ThermoFisher) for 30 min at 37 °C or with 10 μM Rhod-3/AM in the presence of PowerLoad™ and probenecid (R10145, ThermoFisher) for 60 min at room temperature. For Ca^2+^ imaging of the mitochondria, cells were incubated with 2 μM Rhod-FF/AM (R23983, ThermoFisher) for 55 min at 37 °C. For Zn^2+^ indication, cells were incubated with 5 μM FluoZin™-3 AM (F24195, ThermoFisher) (1–7 μM Zn^2+^ cell buffer) for 30 min at 37 °C. For Mg^2+^ indication, cells were incubated with 4 μM Magnesium Green (M3735) (5 mM Mg^2+^ cell buffer) for 30 min at 37 °C. For ROS imaging, cells were incubated with 75 μM carboxy-H2DCFDA (DCF, I36007, ThermoFisher) for 60 min at 37 °C or with 5 μM dihydroethidium (DHE, S0063, Beyotime) for 30 min at 37 °C. For MMP indication, cells were incubated with 0.2 μM TMRM (T668, ThermoFisher) for 30 min at 37 °C. To measure cell viability, cells were incubated with 75 μM propidium iodide (PI, P4170, Sigma) for 10 min at 37 °C. Cells were protected from ambient light during incubation. After staining with dyes, cells were washed and the buffer was replaced with fresh culture medium. For fluorophores with acetoxymethyl (AM) ester, cells were incubated for 10 min to allow complete de-esterification. To test if there is any photoporation of the plasma membrane, 300 μM PI was added to the cell buffer and kept during and after femtoSOC. All green and red fluorescence signals were detected in spectral bands of 500–530 nm and 553–618 nm, respectively.

### Confocal microscopy

For confocal microscopy, 35-mm dishes containing cells were transferred to a custom-built fixator (to minimize mechanical errors during time-lapse microscopy) on the stage of the Nikon upright two-photon confocal microscope (A1 MP + , Nikon). Cells were observed using a 60×, 1.0 NA, water immersion objective lens. Experiments were performed at room temperature. The experimental duration for a single dish of cells was limited within 2 h to keep cells in good conditions. A laser at 488 mm was operated at about 0.1 mW for excitation of Fluo-4, STIM1-YFP, DCF, DHE, and TMRM, and about 3 mW for excitation of flavin’s autofluorescence. For flavin autofluorescence measurements, cells were localized by dishes with a grid at the bottom (Grid-500, ibidi). For Rhod-3 and PI imaging, a laser at 543 nm was operated at about 0.1 mW. Images were acquired for 2.2 μs/pixel with 512 × 512 pixels or 2.4 μs/pixel with 1024 × 1024 pixels and processed by ImageJ for cropping, rotation, and contrast adjustment. Confocal microscopy for *z*-projection imaging was used to observe Orai1’s dynamics, especially Orai1 puncta or clusters. It is important to note that Orai1’s dynamics was not identified if the confocal microscopy plane was in the middle of cells because Orai1 was mostly expressed in the plasma membrane. Therefore, *z*-projection imaging of the cell bottom (membrane surface) was used to provide Orai1 images.

### Materials

2-aminoethyl diphenylborinate (2-APB, D9754, Aldrich), YM-58483 (Y4895, Sigma), thapsigargin (TG, T9033, Sigma), potassium iodide (KI, 221945, Sigma-Aldrich), diphenyliodonium chloride (DPI, 43088, Aldrich), riboflavin (RF, R9504, Sigma), flavin adenine dinucleotide disodium salt hydrate (FAD, F8384, Sigma), rotenone (R8875, Sigma), malonic acid (MA, M1296, Sigma-Aldrich), carbonyl cyanide 4-(trifluoromethoxy)phenylhydrazone (FCCP, C2920, Sigma), MitoTEMPO (M-TEMPO, SML0737, Sigma), TEMPO (214000, Aldrich), α-tocopherol (α-T, T1539, Sigma), and muscimol hydrobromide (G019) were purchased from Sigma-Aldrich. SKF-96365 (ab120280, Abcam) was purchased from Abcam. Tween-20 (A100777, Sangon Biotech) was purchased from Sangon Biotech. DTT (180-8201A, Tanon) was purchased from Tanon. The Ca^2+^-free medium or buffer was composed of 140 mM NaCl, 5 mM KCl, 1 mM MgCl_2_, 10 mM glucose (all from the Sinopharm Chemical Reagent Co., Ltd), 10 mM HEPES, and 20 μM EGTA (both from Sigma). CaCl_2_ was purchased from the Sinopharm Chemical Reagent Co., Ltd. To block SOCs, high-concentration 2-APB was incubated with cells for 30–60 min to provide a moderate block effect since it is a broad-spectrum blocker. SKF-96365, a blocker of voltage gated Ca^2+^ channels, TRPCs, and SOCs and YM-58483, a specific blocker of SOCs, were also used for the inhibition of SOCs. Cells were incubated with SKF-96365 for 5–10 min and YM-58483 for 15 min, respectively, before experiments.

### Protocol of femtoSOC

The two-photon scan with the femtosecond laser (tunable from 690–1040 nm, ~100 fs, 80 MHz, MaiTai DeepSee, Spectra-Physics) in a two-photon microscope was defined by the typical two-photon microscope frame and shared the same galvanometric mirrors of the confocal microscope. It can be inserted into any pre-designed confocal microscopy sequences as a single two-photon microscopy frame. Therefore, it allowed automatic femtoSOC at any pre-defined time and could be achieved by any commercial two-photon microscopy drivers. Once target cells were determined, confocal microscopy was at first stopped. A new confocal microscopy sequence was then defined in the following steps: (1) define a confocal microscopy sequence (the total microscopy duration, frame duration, and number of frames) in the same field of view; (2) define the femtoSOC laser illumination duration and area in the target cell; (3) define the start time slot of each femtoSOC laser illumination, determined by how long femtoSOC should be before and after the laser illumination; (4) define the femtosecond laser parameters of the femtoSOC laser illumination such as the laser wavelength and power; (5) start the confocal microscopy sequence for automatic recording of femtoSOC-induced cellular activity. During confocal microscopy, the femtosecond laser was blocked by an optical shutter. During femtoSOC, the CW lasers for fluorescence excitation were blocked by another optical shutter. The two-photon scan area was defined to be a small region of the target cell (usually 2 × 2 μm^2^) for only one-time use in a short duration (63–500 ms). At 700 nm, the femtosecond laser power was operated at about 1.5 mW at the sample.

### Cell culture

HeLa, HEK293T, and MCF-7 cells were cultured in Dulbecco’s Modified Eagle’s Medium (DMEM, Hyclone). Jurkat T cells were cultured in RPMI 1640 medium (Hyclone). All cells were supplemented with 10% fetal bovine serum (FBS, Gibco) and 1% penicillin/streptomycin (Gibco). To culture primary neurons and astrocytes, the cortex was isolated from C57BL/6 mice postnatal 0 days, digested in 0.25% trypsin (Gibco) for 10 min at 37 °C, and terminated with 10% FBS. After rinsed with phosphate-buffered saline (PBS, Hyclone), the tissue was dissociated and suspended in DMEM supplemented with 10% FBS, followed by filtering with 40-μm strainers (Corning). The cells (~5 × 10^5^ cells/mL) were finally plated in a 35-mm dish (Corning) pre-coated with poly-D-Lysine (Sigma). The medium was replaced with Neurobasal medium (Gibco) supplemented with 2% B27 (Gibco) 3 h after cell plating. The identities of neurons and astrocytes were distinguished based on their morphological features. All cells were cultured on 35-mm dishes at 37 °C in a humidified 5% CO_2_ atmosphere and reached 60%–80% confluent at the time of femtoSOC.

### Plasmid construction and transfection

GCaMP6s, CEPIA3mt, and G-CEPIA1ER used in this study were generous gifts from Dr. Franck Polleux. NES-jRGECO1a (Addgene plasmid #61563), G-GECO1-Orai1 (Addgene plasmid #73561), ERK2-GFP (Addgene plasmid #37145), Orai1-YFP (Addgene plasmid #19756), and STIM1-YFP (Addgene plasmid #18857) were purchased from Addgene. Lck-GCaMP5G was cloned and purchased from GeneChem, Shanghai. Orai1-mCherry was cloned and purchased from BioFeng, Shanghai. The plasmids encoding Orai1 (Genechem) were constructed by subcloning human Orai1 into GV417 vector (CMV-MCS-IRES-Cherry-SV40-Neomycin) between *Nhe*I and *Bam*HI. RNA interference of Orai1 or TRPC1 was performed using the plasmid encoding Orai1 or TRPC1 shRNA (Genechem), respectively, which was constructed by subcloning the target sequence into GV298 vector (U6-MCS-Ubiquitin-Cherry-IRES-puromycin) between *Age*I and *Bam*HI. The target sequence for Orai1 shRNA was TGTCCTCTAAGAGAATAAG, the sequence for TRPC1 shRNA was ATCGTTACTTGACTTCCAT, and the control sequence was TTCTCCGAACGTGTCACGT.^44^ HeLa cells were transiently transfected with the plasmids through jetPRIME (Polyplus-transfection) according to the manufacturer’s instructions. In brief, ~3 × 10^5^ cells were seeded on a 35-mm dish in 2 mL of DMEM supplemented with 10% FBS and 1% penicillin/streptomycin 24 h prior to the transfection, which enabled cells to reach 60%–80% confluent at the time of the transfection. Then, 1 μg plasmids were dilute into 200 μL buffer, followed by adding with 2 μL jetPRIME and incubation for 10 min at room temperature. The mixed buffer was added into the dish drop wise and replaced with refresh culture medium after 4–5 h. For femtoSOC experiments, shRNA-transfected cells were prepared 24–48 h after the transfection, while Orai1-transfected cells were prepared 72 h after the transfection.

### Orai1 knockout by CRISPR

A set of three CRISPR sgRNA sequences was designed to target the *Orai1* gene (NCBI Gene identification number 84876, target sequence 3’-CTGGCCGATCCAGTCCGGGTAGG-5’ and 5’-TAAAGCCTCCAGCCGGACCTCGG-3’ in the exon 1 of the *Orai1* precursor messenger RNA and 5’-CGCTGACCACGACTACCCACCGG-3’ in the exon 2 of the *Orai1* precursor messenger RNA (GenBank accession number NM_032790.3)). The sgRNA sequences were used in conjunction with a Cas9-coding plasmid, while Lenti-U6-sgRNA-EFS-CRISPR-PGK-cherry was transformed from lentiCRISPR v2 (Addgene, Plasmid#52961) to generate Orai1-KO single-cell clones. The virus was enriched, filtered, and transfected to HeLa cells. The mCherry-positive cells were sorted by flow cytometry and cloned. The effectiveness of the knockout was tested by PCR-Sanger sequencing. The results show that the sequences 5’-GGCGG-3’ in the Orai1-KO cells were absent compared to WT cells, indicating that they are a homozygote of the *Orai1* gene knockout. Furthermore, the absent expression of *Orai1* was verified by western blotting and the result was shown in Fig. 3f. The technology was studied from a recent work.^45^

### STIM1 knockout by CRISPR

A set of three CRISPR sgRNA sequences was designed to target the *STIM1* gene (NCBI Gene identification number 6786, target sequence 5’-TATGCGTCCGTCTTGCCCTGTGG-3’ in the exon 1 and 5’-TATCCAGAACCGTTACTCCAAGG-3’, 5’-GGTGTCTATCGTTATTGGTGTGG-3’ in the exon 6 of the *STIM1* precursor messenger RNA (GenBank accession number NM_003156.3)). This knockout process was identical to that for Orai1-KO cells. The results of PCR-sanger sequencing show that the sequences 5’-GTGTGGGCGGCTGCTGGTTTGCCTATATCCAGAACCGTTAC-3’ in STIM1-KO cells were absent compared to WT cells, indicating that they are a homozygote of the *STIM1* gene knockout. Furthermore, the absent expression of *STIM1* was verified by western blotting and the result was shown in Fig. 4i. This work was assisted by Shanghai Huzbio Biotechnology Co., Ltd.

Orai1/2/3-KO cells were a gift from the lab of Prof. Mohamed Trebak from Penn. State. Uni. The absent expression of STIM1 was verified by western blotting and the result was shown in Supplementary information, Fig. S7.

### Western blotting and immunofluorescence microscopy

Cells were dissected and homogenized in lysis buffer (RIPA: protease inhibitor cocktail = 100:1). Whole cell lysates were analyzed in a western blot experiment according to standard protocols. Antibodies for immunoblotting were as follows: Orai1 rabbit polyclonal antibody (13130-1-AP, Proteintech, 1:100 dilution), rabbit anti-ORAI1 (O8264, Sigma, 1:1000 dilution), TRPC1 rabbit polyclonal antibody (19482-1-AP, Proteintech, 1:500 dilution), GAPDH mouse monoclonal antibody (60004-1-Ig, Proteintech, 1:10,000 dilution), Peroxidase AffiniPure goat anti-mouse IgG (H + L) (115-035-003, Jackson ImmunoResearch, 1:10,000 dilution), Peroxidase AffiniPure goat anti-rabbit IgG (H + L) (111-035-003, 1:10,000 dilution, Jackson ImmunoResearch). Proteins were visualized using enhanced chemiluminescence. The images were acquired by Tanon 4200SF (Tanon). Immunofluorescence microscopy of eIF4E-p was performed 24 h after femtoSOC. Cells were fixed with 5% paraformaldehyde and permeabilized with 0.5% Triton X-100 (1848549B, Life Technologies). The cells were then incubated with an anti-eIF4E antibody (phosphor S209, 1 μg/mL, ab76256, Abcam) overnight at 4 °C. The sample was washed with PBS for 5 min and incubated with diluted secondary antibody anti-Rabbit IgG (H + L) (Alexa Fluor 488, 2 μg/mL, ab150077, Abcam) for 1.5 h at room temperature.

### Cell viability measurements

To measure cell viability and proliferation rate after femtoSOC, dishes with a grid at the bottom (Grid-500, ibidi) were adopted to locate cells for femtoSOC. In brief, all cells within the grid of Grid-500 dishes were scanned by the femtosecond laser and then cultured at 37 °C in a humidified 5% CO_2_ atmosphere. After 6, 15, or 25 h, cells were stained with PI and observed by the confocal microscope to record the number of viable and dead cells.

### Preparation of mouse brain slices

Brain slices of C57BL/6 mice were prepared by cutting brain tissue from an area around the hippocampus after sacrificing the mice immediately (VT1200S, Leica). The brain slices were 300-μm thick and incubated in the perfusion chamber at room temperature in an atmosphere of 95% O_2_ and 5% CO_2_ with artificial cerebrospinal fluid (ACSF) which contains 125 mM NaCl, 1.25 mM KCl, 25 mM NaHCO_3_, 1.25 mM KH_2_PO_4_, 25 mM D-Glucose, 2 mM CaCl_2_, 1 mM MgCl_2_, supplemented with 2 mM Na-pyruvate and 0.5 mM L-ascorbic acid during loading of Cal-520/AM (21130, AAT Bioquest, incubated at 6 μM for 30 min, followed by washing for 30 min) and observed with a multiphoton confocal microscope (SP8, Leica).

### Preparation of intact living mouse brains

For in vivo experiments, 6- to 9-week-old C57BL/6 mice were anesthetized with 1% pentobarbital. After the surgical level of anesthesia was reached, the skin and skull (~1 mm diameter) above the desired brain area were removed in sequence. Then, dental cement was used to adhere the brain to the custom-made stage of the microscope and to form a recording chamber above the imaging window in order to keep the brain moist with ACSF. To introduce the calcium indicator to the targeted area of the brain, a glass pipette with a 20-μm diameter open tip filled with 1 μL Cal-520/AM (~0.6 mM) was inserted into the cortex (~400–450 μm below the cerebral dura mater). The fluorophore was injected at a speed of 40 nL/min. 1.5 h after the injection, the brain was observed by the upright two-photon confocal microscope with a 25×, 0.95 NA, water immersion objective lens (SP8, Leica). To activate a randomly selected neuron, the femtosecond laser light was scanned in a small area (~2 × 2 μm^2^) of the neuron as a single frame of 0.5 s at a wavelength of 700 nm and an average laser power of ~7 mW (measured before the sample). All animal experiments were performed in compliance with the Guide for the Care and Use of Laboratory Animals and the protocols approved by the Animal Study Committee of the School of Biomedical Engineering at Shanghai Jiao Tong University and the School of Life Science, at the University of Science and Technology of China.

### Statistics

As a cautious and strict standard, the Ca^2+^ rise of femtoSOC-excited cells was defined by a threshold, 1.2 times of the fluorescence baseline, indicated by Fluo-4. The data were presented as the means ± standard error of the mean (SEM) and analyzed with GraphPad Prism 7 software throught the one-tailed paired *t*-test except where stated otherwise. The confidence interval for *t-*tests was set as 95%.

## Supplementary information

Supplementary information, Fig. S1

Supplementary information, Fig. S2

Supplementary information, Fig. S3

Supplementary information, Fig. S4

Supplementary information, Fig. S5

Supplementary information, Fig. S6

Supplementary information, Fig. S7

Supplementary information, Fig. S8

Supplementary information, Fig. S9

Supplementary information, Fig. S10

Supplementary information, Fig. S11

Supplementary information, Fig. S12

Supplementary information, Fig. S13

Supplementary information, Video legend

Supplementary information, Video. S1

Supplementary information, Video. S2
