# Supplementary material for: Direct control of store-operated calcium channels by ultrafast laser
Source: Cell Res. 2021 Jan 19;31(7):758–72. doi: 10.1038/s41422-020-00463-9 (PMC8249419; doi:10.1038/s41422-020-00463-9)
Supplement: Supplementary file 1 — Supplementary information, Fig. S1 [file 41422_2020_463_MOESM1_ESM.pdf]

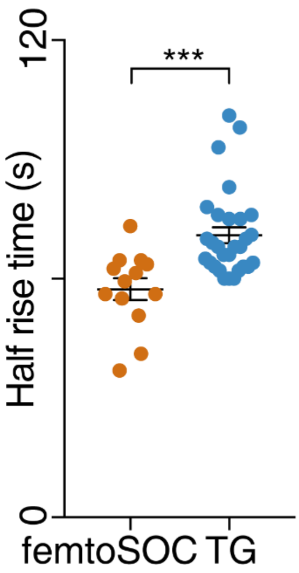

Fig. S1. Half rise time of Ca^{2+} in femtoSOC-excited cells and TG-treated cells (indicated by Fluo-4 AM).
