# Supplementary material for: Direct control of store-operated calcium channels by ultrafast laser
Source: Cell Res. 2021 Jan 19;31(7):758–72. doi: 10.1038/s41422-020-00463-9 (PMC8249419; doi:10.1038/s41422-020-00463-9)
Supplement: Supplementary file 2 — Supplementary information, Fig. S2 [file 41422_2020_463_MOESM2_ESM.pdf]

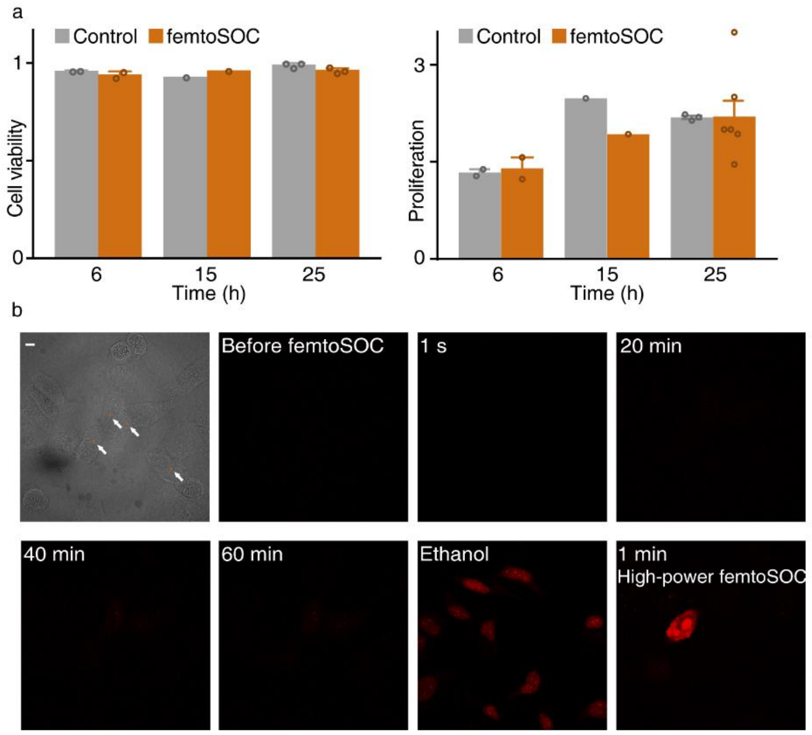

Fig. S2. Viability and membrane integrity of femtoSOC-excited cells. (**a**) Cellular viability rate in the presence of PI (300 μM) and cellular proliferation rate after femtoSOC in comparison with the control group (n = 2, 1, and 3 independent experiments in the 6, 15 and 25 h groups). (**b**) Cellular membrane integrity in the presence of PI (300 μM) in comparison with the positive control (damaged by alcohol or by a high laser power of 50 mW for 1 s) (n = 15 cells).
