# Supplementary material for: Direct control of store-operated calcium channels by ultrafast laser
Source: Cell Res. 2021 Jan 19;31(7):758–72. doi: 10.1038/s41422-020-00463-9 (PMC8249419; doi:10.1038/s41422-020-00463-9)
Supplement: Supplementary file 3 — Supplementary information, Fig. S3 [file 41422_2020_463_MOESM3_ESM.pdf]

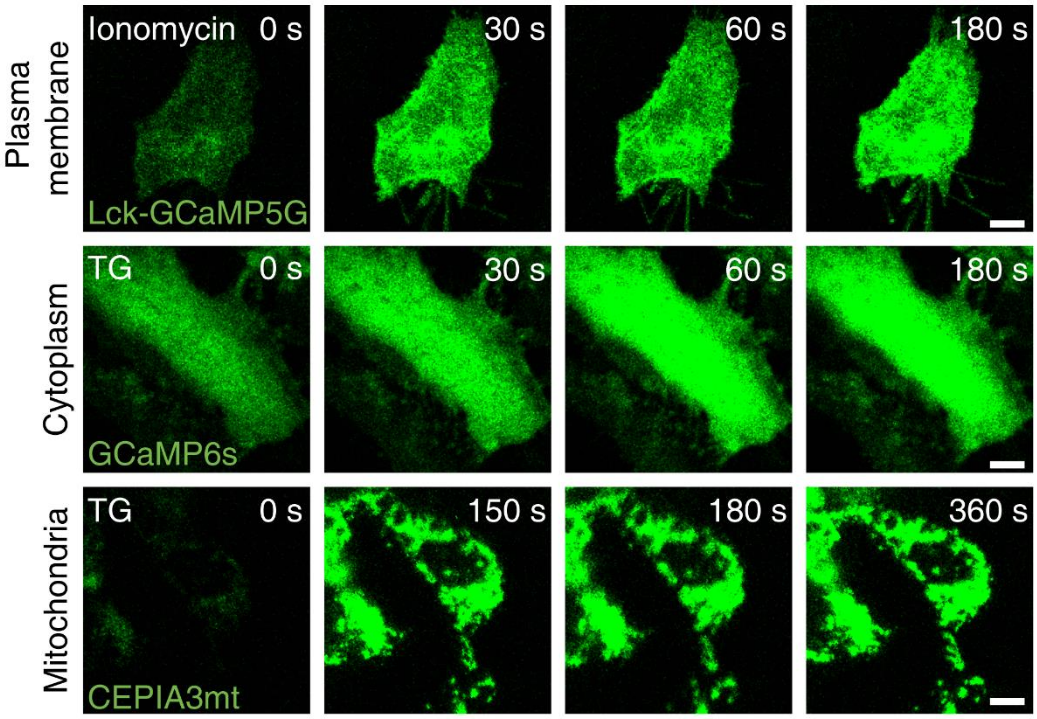

Fig. S3. Cells transfected with Lck-GCaMP5G, GCaMP6s, and CEPIA3mt to visualize the localized Ca^{2+} level in the cell membrane, cytoplasm, and mitochondria, respectively (n = 60 cells). The response of those fluorescent proteins to Ca^{2+} was verified by ionomycin or TG treatment which all increased after the treatment as expected. Scale bars: 10 μm.
