# Supplementary figures and images for: Direct control of store-operated calcium channels by ultrafast laser
Source: Cell Res. 2021 Jan 19;31(7):758–72. doi: 10.1038/s41422-020-00463-9 (PMC8249419; doi:10.1038/s41422-020-00463-9)

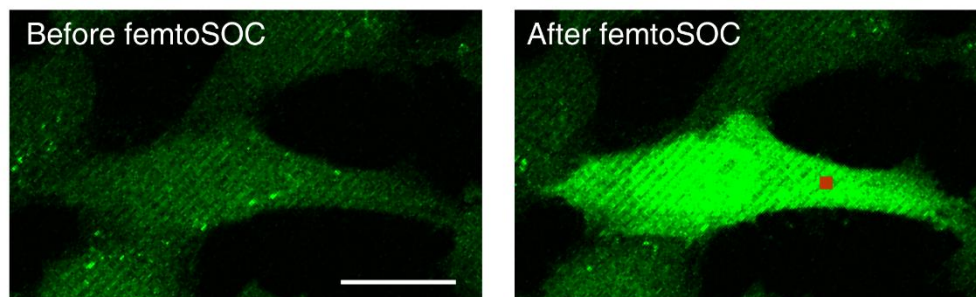

**Fig. S4. femtoSOC-induced  $\text{Ca}^{2+}$  influx in cells incubated with 20  $\mu\text{M}$  Ryanodine.**

Supplement: Supplementary file 4 — Supplementary information, Fig. S4 [file 41422_2020_463_MOESM4_ESM.pdf]

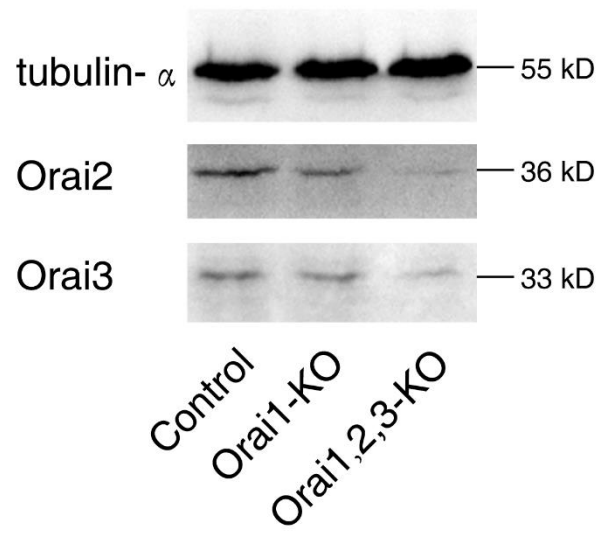

**Fig. S7. Western blot of Orai2 and Orai3 in Orai1-KO and Orai1,2,3-KO cells.**

Supplement: Supplementary file 7 — Supplementary information, Fig. S7 [file 41422_2020_463_MOESM7_ESM.pdf]

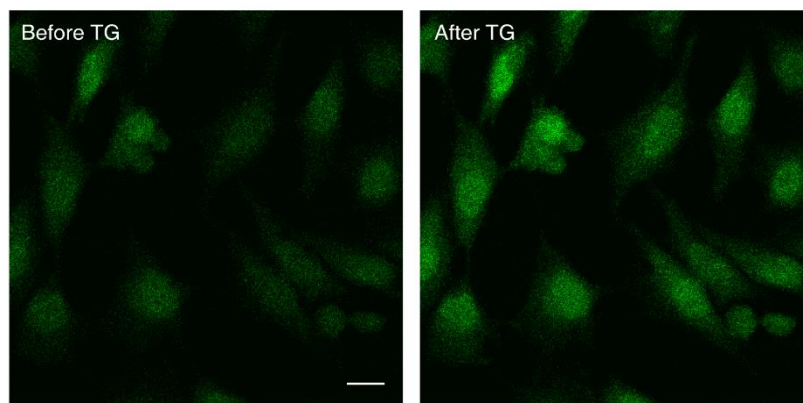

**Fig. S9.** Cells with Fluo-4 in the presence of 50 mM KI before and after TG treatment. Scale bar:

20  $\mu$ m.

Supplement: Supplementary file 9 — Supplementary information, Fig. S9 [file 41422_2020_463_MOESM9_ESM.pdf]

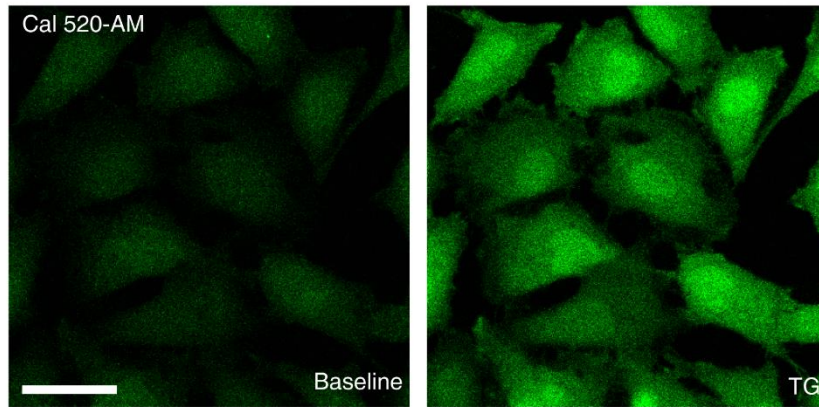

**Fig. S10.** Cells with DTT treatment, showing no effect on  $\text{Ca}^{2+}$  influx by TG. Scale bar: 20  $\mu\text{m}$ .

Supplement: Supplementary file 10 — Supplementary information, Fig. S10 [file 41422_2020_463_MOESM10_ESM.pdf]

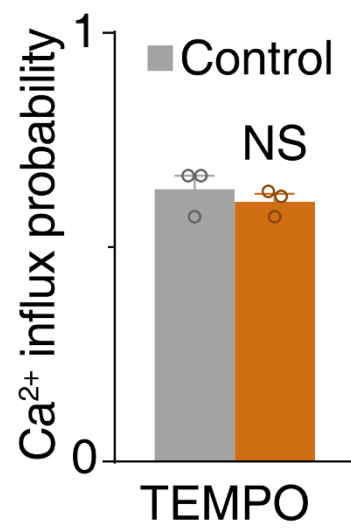

**Fig. S12.**  $\text{Ca}^{2+}$  influx probability of cells treated with 1 mM TEMPO.

Supplement: Supplementary file 12 — Supplementary information, Fig. S12 [file 41422_2020_463_MOESM12_ESM.pdf]

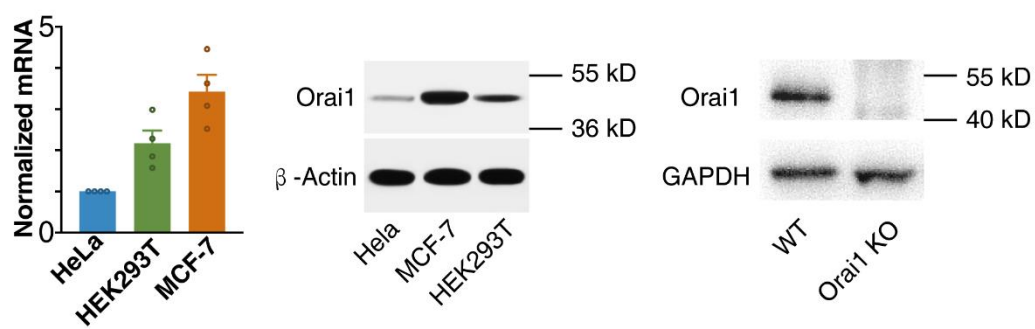

**Fig. S13. Orai1 level by qPCR and Western Blot in different cell lines.**

Supplement: Supplementary file 13 — Supplementary information, Fig. S13 [file 41422_2020_463_MOESM13_ESM.pdf]
