# Supplementary material for: Direct control of store-operated calcium channels by ultrafast laser
Source: Cell Res. 2021 Jan 19;31(7):758–72. doi: 10.1038/s41422-020-00463-9 (PMC8249419; doi:10.1038/s41422-020-00463-9)
Supplement: Supplementary file 5 — Supplementary information, Fig. S5 [file 41422_2020_463_MOESM5_ESM.pdf]

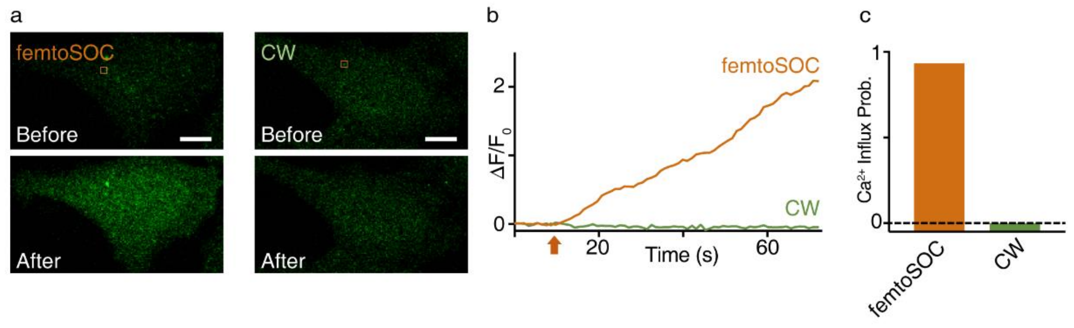

Fig. S5. Absence of Ca^{2+} influx in cells excited by CW laser light. (**a**, **b**) Comparison in cellular response between femtoSOC and CW laser excitation (both 760 nm). The CW laser light with 10 times higher laser power did not even activate any Ca^{2+} influx. (**c**) Comparison in Ca^{2+} influx probability between femtoSOC and CW laser excitation (n = 15 cells). Scale bars: 10 μm.
