# Supplementary material for: Direct control of store-operated calcium channels by ultrafast laser
Source: Cell Res. 2021 Jan 19;31(7):758–72. doi: 10.1038/s41422-020-00463-9 (PMC8249419; doi:10.1038/s41422-020-00463-9)
Supplement: Supplementary file 6 — Supplementary information, Fig. S6 [file 41422_2020_463_MOESM6_ESM.pdf]

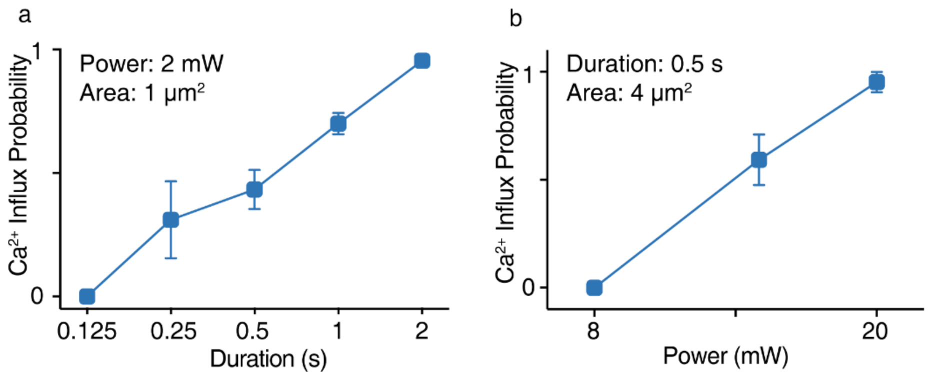

Fig. S6. Ca^{2+} influx probability of cells. (**a**) Increasing probability with increasing femtoSOC laser illumination duration. (**b**) Increasing probability with increasing femtoSOC laser illumination power (n = 2 – 4 independent experiments for each point; n = 15 – 21 cells in each independent experiment).
