# Supplementary material for: Direct control of store-operated calcium channels by ultrafast laser
Source: Cell Res. 2021 Jan 19;31(7):758–72. doi: 10.1038/s41422-020-00463-9 (PMC8249419; doi:10.1038/s41422-020-00463-9)
Supplement: Supplementary file 8 — Supplementary information, Fig. S8 [file 41422_2020_463_MOESM8_ESM.pdf]

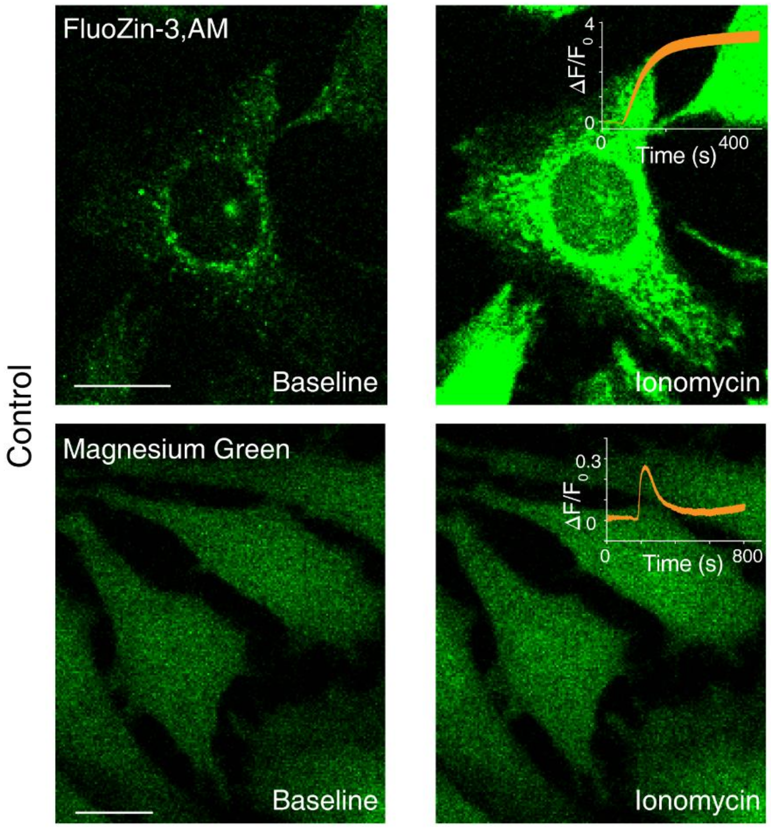

Fig. S8. Control tests of FluoZin-3, AM and Magnesium Green to indicate Zn^{2+} and Mg^{2+}, respectively. Cells loaded with FluoZin-3, AM and Magnesium Green were treated with ionomycin (10 μM) and Zn^{2+} (1 – 7 μM) and Mg^{2+} (1 – 5 mM). The fluorescence increase suggests the influx of Zn^{2+} and Mg^{2+} by ionomycin. Scale bars: 20 μm.
