# Supplementary material for: Direct control of store-operated calcium channels by ultrafast laser
Source: Cell Res. 2021 Jan 19;31(7):758–72. doi: 10.1038/s41422-020-00463-9 (PMC8249419; doi:10.1038/s41422-020-00463-9)
Supplement: Supplementary file 11 — Supplementary information, Fig. S11 [file 41422_2020_463_MOESM11_ESM.pdf]

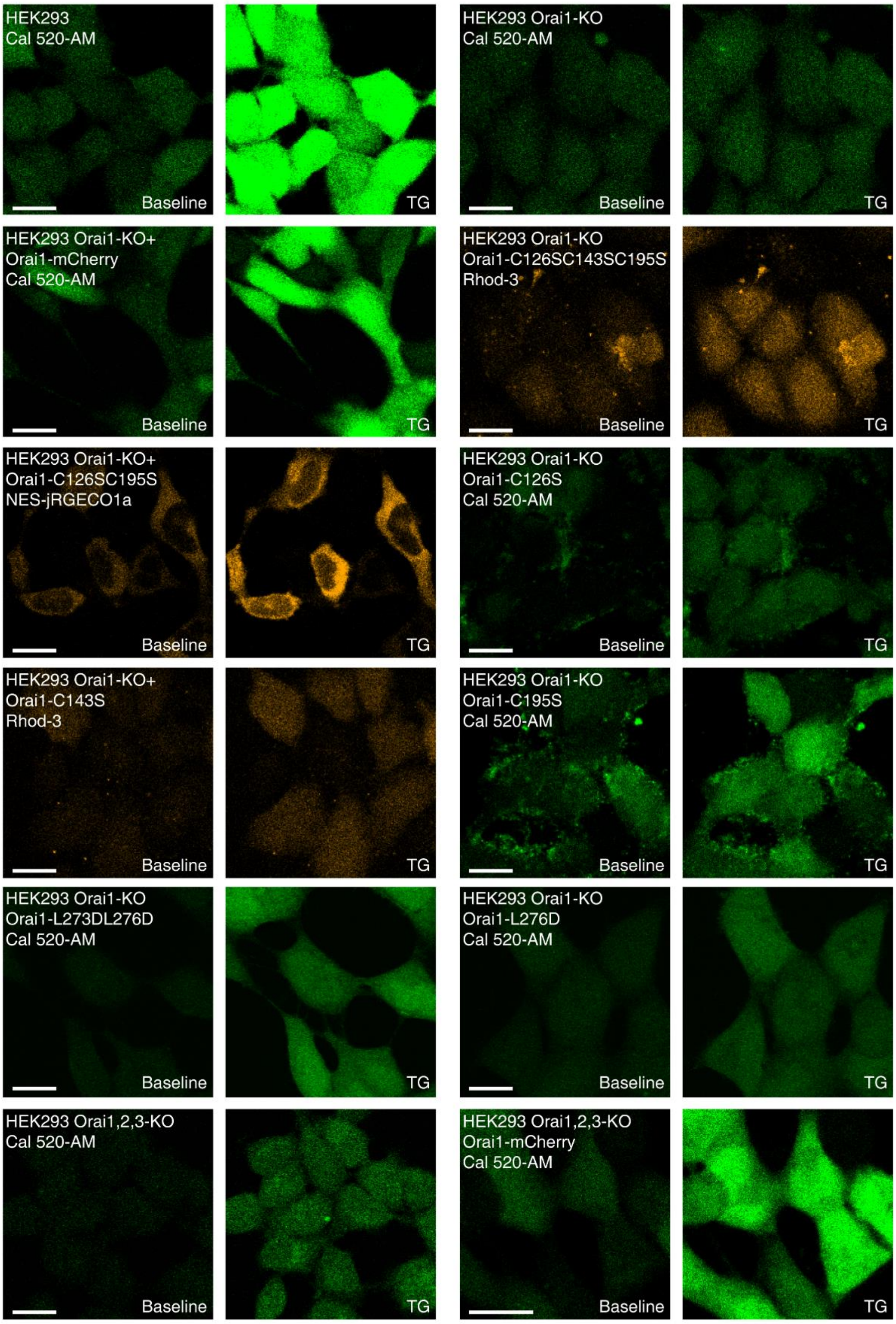

Fig. S11. Cells with different Orai1 mutants tested by TG treatment. Significant Ca^{2+} influx was found in all cells with different Orai1 mutants, indicating that Orai1 is important in femtoSOC. No Ca^{2+} influx was found in Orai1-KO cells and Orai1,2,3-KO cells. Scale bars: 20 μm.
