# Supplementary material for: Direct control of store-operated calcium channels by ultrafast laser
Source: Cell Res. 2021 Jan 19;31(7):758–72. doi: 10.1038/s41422-020-00463-9 (PMC8249419; doi:10.1038/s41422-020-00463-9)
Supplement: Supplementary file 14 — Supplementary information, Video legend [file 41422_2020_463_MOESM14_ESM.pdf]

Video S1. femtoSOC of a single target neuron in a fresh brain slice. Note a single frame for femtoSOC in a micro-region could be found during the imaging.

Video S2. Two-time femtoSOC of a single target neuron in a living intact mouse brain. Note a single frame for femtoSOC in a micro-region could be found during the imaging.
